# Adaptive Localizing Region-Based Level Set for Segmentation of Maxillary Sinus Based on Convolutional Neural Networks

**DOI:** 10.1155/2021/4824613

**Published:** 2021-11-11

**Authors:** Xianglong Qi, Jie Zhong, Shengjia Cui

**Affiliations:** ^1^Liaoning Huading Technology Co., Ltd., Shenyang, Liaoning 110167, China; ^2^JiangSu PangPu Network Technology Co., Ltd., JiangSu, China; ^3^Baidu.com Times Technology (Beijing) Co., Ltd., Beijing, China

## Abstract

In this paper, we propose a novel method, an adaptive localizing region-based level set using convolutional neural network, for improving performance of maxillary sinus segmentation. The healthy sinus without lesion inside is easy for conventional algorithms. However, in practice, most of the cases are filled with lesions of great heterogeneity which lead to lower accuracy. Therefore, we provide a strategy to avoid active contour from being trapped into a nontarget area. First, features of lesion and maxillary sinus are studied using a convolutional neural network (CNN) with two convolutional and three fully connected layers in architecture. In addition, outputs of CNN are devised to evaluate possibilities of zero level set location close to lesion or not. Finally, the method estimates stable points on the contour by an interactive process. If it locates in the lesion, the point needs to be paid a certain speed compensation based on the value of possibility via CNN, assisting itself to escape from the local minima. If not, the point preserves current status till convergence. Capabilities of our method have been demonstrated on a dataset of 200 CT images with possible lesions. To illustrate the strength of our method, we evaluated it against state-of-the-art methods, FLS and CRF-FCN. For all cases, our method, as assessed by Dice similarity coefficients, performed significantly better compared with currently available methods and obtained a significant Dice improvement, 0.25 than FLS and 0.12 than CRF-FCN, respectively, on an average.

## 1. Introduction

Nasal diseases are growing common for individuals with serious impacts on daily life. In America, there are about 16% adults suffering from this trouble, and there are 100 million in China [1]. Acute sinusitis is simple for management with antibiotic drugs [2]. However, for chronic one, functional endonasal sinus surgery (FESS) may be the only solution of relief. FESS contains some processes: maxillary sinus fenestration, ethmoid sinus resection, etc. [3]. It is found that a lot of risks may appear in surgery due to great variations of nasal anatomy, where optical nerves and carotids have higher possibility to be affected [4]. Segmentation and the subsequent quantitative assessment of lesions in medical images provide valuable information for the analysis of diseases, and quantitative imaging can reveal clues about lesion characteristic and anatomical structure. For example, preoperative computed tomography (CT) affords radiologists the opportunity to prospectively identify anatomic variant that predispose patients to major surgical complications [5]. A real-time surgical navigation has joined in FESS [6]. To make FESS safer, surgeons use navigation systems that register a patient to his/her CT scan and track the position of tools inside the patient [7].

There are growing evidences that the qualification of sinuses increases insights into functional outcomes and requires accurate segmentation which is a challenging task for a number of reasons. The heterogeneous appearance of lesions including large variability in location, size, shape, and frequency makes it difficult to design effective segmentation rules. Although the most accurate segmentation results can be obtained through manual delineation by an experienced expert, it costs plenty of tedious time, nearly, 8–10 hours per case of one patient [8]. Besides, the expertise decides whether a particular region is a part of the segmentation object. In order to understand the complexity of maxillary sinus, it is necessary to conduct wide studies to gain statistical pattern for drawing conclusions. Therefore, a more accurate, automatic segmentation algorithm has been required as soon as possible.

**Figure 1:**
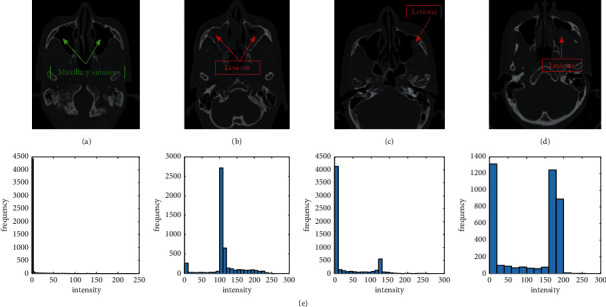
Inhomogeneous appearance of maxillary sinus shows challenges in research of segmentation. (a) A pair of healthy cavity and (b–d) lesions, sizes, locations, and shapes which are diffused and reveal the wide spatial distribution. Failed segmentation is more likely caused by incorrect noisy information. (e) The average of the normalized intensity histograms of corresponding CT images in maxillary sinus. Different distributions can be observed easily and demonstrate difficulty for most popular methods.

Figure 1 illustrates some of the potential challenges when proposing a computational approach for the task of automatic maxillary sinus segmentation. The figure demonstrates intensity statistics of great difference and shows examples of lesions in the cavity of maxillary sinus. Lesions can be found at multiple sites, with different shapes and sizes which misguide curve evolution as a result of high gradient noisy areas. Meanwhile, it is generally difficult to derive statistical prior knowledge to set up a similar description for shape of maxillary sinus due to common anatomical abnormality [9]. Ideally, an acceptable solution is able to adjust itself to foreground by learning from potential features in a few of examples.

Level set is popular as a curve evolution application, especially for medical image segmentation [10]. Compared with active contour methods based on points' motion, it has the ability to handle image noise, intensity heterogeneity, and discontinuous object boundaries. Level set methods include models based on edge [10–12] or region [13–15]. Edge-based models are sensitive to noise and objects with incomplete boundaries or low contrast texture. Region-based ones study spatial statistics of interesting regions to find global minima of energy functional but lack competence in details. While, in many examples of great heterogeneous foreground and background, a local region model has a better performance than the global one [16–20]. Hybrid models are superior, defining an energy functional with local and global constraints to obtain a more robust segmentation with little sensitive initialization.

In level set works, the choice of functional control parameters is puzzled for a long time. Li et al. [21] give proof that an inappropriate definition may lead to an inferior segmentation regardless of initialization. As Lankton et al. described [16], parameters have close relationships with direction and speed of active contour according to specific texture feature. A slight change in the group of parameters even produces entirely different results. Therefore, the fixed parameters cannot survive under different spatial distributions of intensity in maxillary sinus with lesion. Some researches tend to test an optional value over a series of training set for the entire database of images, but usually, new images may require additional experiments to find the best-fitted parameters. As a result, choosing a fixed set of parameters by trial is a time-consuming and laborious process. In addition, most users do not have enough experience to tune a large number of parameters optimally. For this reason, an adaptive strategy is absolutely desirable. If, in the process of segmentation, parameters could be adjusted dynamically based on specific texture, the approach has a higher chance of providing a more accurate segmentation.

Some papers present an algorithm to estimate parameters for energy functional before segmentation. Li et al. [21] introduce a method to adjust parameters of the level set model according to classification of pixels with intensity. Unfortunately, the value is initialized once at the beginning of work and remains constant all the time, which causes a poor segmentation especially for the object with great variant lesions. Oliviera et al. [22] provide a mechanism to evaluate parameters from analysis of the training set which also can be reused in new images. It is obviously inappropriate for sinus cavity with highly diverse lesions. Baillard et al. [23] devise a solution for the problem of spatial heterogeneity and take full considerations for every point position in every iteration. That is, a point on the contour at first determines its status. If it belongs to the object, it should locally extend outwards. And, if not, it should move in the opposite direction. This classification depends on Gaussian and the shifted Rayleigh statistical distribution models via training set by maximizing the posterior probability. However, because of the diversity of lesion, its prediction accuracy of position would be doubted and discussed depending on the conventional machine learning method. Consequently, [21–23] are likely to perform unacceptably for highly diverse datasets and need more optimizations.

At the same time, deep learning techniques have appeared as a powerful alternative for supervised learning applications such as classification, detection, and other areas [24–27]. Convolutional neural network (CNN) [28, 29] can learn highly discriminative features and have been widespread with predominance on a variety of problems including medical imaging. Ciresan et al. [30] adopt classical CNN for the segmentation of the neural membrane. Obviously, this strategy has two drawbacks. At first, it costs so much computing time since the network must be run separately for each patch, and there is a lot of redundancy due to overlapping patches. In addition, there is a trade-off between localization accuracy and the use of context. For more accurate localization, Ronneberger et al. give a more elegant architecture U-Net derived from fully convolutional network (FCN) [31], which consists of a contracting path to capture context and a symmetric expanding path. Although the algorithm gains a certain achievement, the result of the object specifically in edge is too coarse to be accepted for medical image segmentation. Kamnitsas et al. [32] incorporate conditional random field (CRF) into FCN. Details of segmentation are refined by fuzzy classification based on relations between position and class of pixels in future. It is a pity that this method suffers from noises seriously. Assaf et al. [33] propose to predict the position of active contour with CNN. As this method focuses on entire contour status on average, it cannot deal with the irregular shape of object particularly. These methods are proved successful in computer vision applications on medical images. The idea of CNN strengthens knowledge of significant features on large scale of the training set, while a big challenge for appearance or texture with great distribution in space has come up with maxillary sinus segmentation. It is impossible to set up a class-balance training set to describe all possible cases with lesion.

In this paper, we present a novel improvement of the level set segmentation using the convolutional neural network. Our method is a multistage process. A convolutional neural network is used to identify the location of a point on the zero level set contour. If the point has high probability close to lesion area, its speed can be compensated to a certain extent based on output probabilities of CNN. If not, we believe it has arrived in the target area. This interactive procedure happens when minimizing the cost functional over iterations of the level set. It is unnecessary for a more accurate initial contour at the beginning of segmentation. Contrary to current hybrid level set frameworks, our method has little dependence on special initialization and does not include any assumptions about sinuses and lesion characteristics. Therefore, our proposed algorithm has the strong adaptiveness in diverse datasets of maxillary sinus with heterogeneous lesion that include plenty of noise and abnormal anatomical structure.

To the best of our knowledge, this is the first use of CNN to identify location of points on the zero level set with some magnitude of speed compensation, avoiding energy functional from being trapped into local minima and resulting in a generalized segmentation solution than methods available to date.

## 2. Materials and Methods

### 2.1. Energy Models

We used two different energy models to extensively evaluate our proposed adaptive localizing region-based segmentation algorithm: uniform modeling energy (UM) and mean separation energy (MS). In this paper, the innovative method is optimized from local version of the level set [16], and energy is given as follows:

(1) $$\begin{array}{c} E\left(\phi \right)=\int_{\Omega_{x}}\delta \phi \left(x\right)\int_{\Omega_{y}}B\left(x,y\right)\cdot F\left(I\left(y\right),\phi \left(y\right)\right)\text{d}y\text{d}x+\mu \int_{\Omega_{y}}\delta \phi \left(y\right)\left|▽\phi \left(y\right)\right|\text{d}y, \end{array}$$

where *x* and *y* denote the position of specific pixel located in the coordinate of current image, *B*(*x*, *y*) represents local regions centering at every point on the contour, such as a rectangular or circle, and the function *F* is a generic internal energy measure used to describe local adherence to a given model at each point along the contour. In order to keep the curve smooth, we add a regularization term as is commonly done. Therefore, the UM or MS model is selected as possible candidates for *F* to be implemented in our proposed method. By taking the first variation of (1) with respect to *ϕ*, we obtain the following evolution equation:

(2) $$\begin{array}{c} \frac{\partial \phi }{\partial t}\left(x\right)=\delta \phi \left(x\right)\int_{\Omega_{y}}B\left(x,y\right)\cdot {▽}_{\phi \left(y\right)}F\left(I\left(y\right),\phi \left(y\right)\right)\text{d}y+\mu \delta \phi \left(x\right)\text{div}\left(\frac{▽\phi \left(x\right)}{\left|▽\phi \left(x\right)\right|}\right). \end{array}$$

#### 2.1.1. Uniform Modeling Energy

A well-known example of an energy that uses a constant intensity model is the Chan-Vese energy [13], which we will refer to as the uniform modeling energy described as

(3) $$\begin{array}{c} F_{UM}=\int_{\Omega_{y}}\lambda_{1}H_{\phi \left(y\right)}{\left(I\left(y\right)-u\right)}^{2}+\lambda_{2}\left(1-H_{\phi \left(y\right)}\right){\left(I\left(y\right)-v\right)}^{2}\text{d}y. \end{array}$$

The energy model divides region of interest (ROI) into foreground and background. *u* and *v* represent their intensity means, respectively. Set Ω as a bounded subset in *R*^2^ and *I*(*y*) as the coordinated of a point on image *I*. Let *ϕ*(*y*) be a signed distance map and *H*_*ϕ*(*y*)_ be the foreground region. A local version of the UM model can be used by replacing *u* and *v* with their local versions, *u*_*x*_ and *v*_*x*_, to represent the local means of a region divided into exterior and interior surrounding each contour point, according to equation (4). *λ*_1_ and *λ*_2_ are parameters that have impactions on speed direction and magnitude which have close relationship with the texture of ROI. The related derivation process could be provided in [13]:

(4) $$\begin{array}{c} F_{UM}=\mu \int_{\Omega_{y}}\delta \phi \left(y\right)\left|▽\phi \left(y\right)\right|\text{d}y+\lambda_{1}\int_{\Omega_{y}}{\left|I\left(y\right)-u_{x}\right|}^{2}H_{\phi }\left(y\right)\text{d}y+\lambda_{2}\int_{\Omega_{y}}{\left|I\left(y\right)-v_{x}\right|}^{2}\left(1-H_{\phi }\left(y\right)\right)\text{d}y. \end{array}$$

#### 2.1.2. Mean Separation Model

The mean separation model is first proposed by Yezzi et al. [34] which we refer to as mean separation energy:

(5) $$\begin{array}{c} F_{MS}=\int_{\Omega_{y}}{\left(u-v\right)}^{2}. \end{array}$$

This energy functional arrives in minima when foreground and background regions have maximally separate mean intensities. There is a strong assumption that the object and its background have the largest difference of intensity. There is no restriction on how well regions are modeled by *u* and *v*. In our method, we design the local version of *F* as

(6) $$\begin{array}{c} F_{MS}={\left(u_{x}-v_{x}\right)}^{2}. \end{array}$$

By substituting the derivative of *F*_*MS*_ into (2), we obtain the local region based on speed flow. This allows the MS model to find image edges effectively without considering the uniformity of global internal or external regions.

### 2.2. The Proposed Method

**Figure 2:**
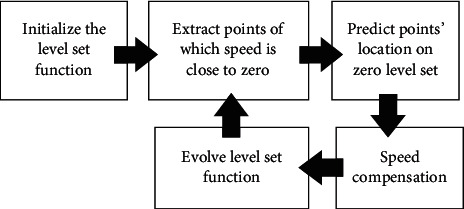
Pipeline of the proposed method with speed compensation.

The proposed method contains an interactive hybrid of the localizing level set and CNN, illustrated in Figure 2, which estimates the probabilities of points' location and helps the energy functional escape from local minima effectively. From borrowed spirits from previous work [35], we initialize the level set model with a contour surrounding the segmentation object. In general, the contour should move to the target as the required speed as level set functional. However, as the result of noisy disturb, the moving contour may be trapped in the local noises. Therefore, we predict the real target possible location and suggest new speed direction and value to accelerate the moving contour moving state evolving more reasonably.

#### 2.2.1. Speed Compensation

As introduced in Section 1, difficulties in the segmentation of maxillary sinus are lesions with uncertain positions, shapes, and intensities. Certainly, a healthy one can be dealt with readily. Holding higher gray value, lesions are easy to frustrate evolution of the initial contour that hardly arrives in the target. In our optimization, we propose a solution of speed compensation to resolve this problem. That is, if speed of a point on the contour is detected close to zero, a devised convolutional neural network is required to identify features of its local region. For more accurate result, we train it on a large scale of the training set. About details, we will discuss it later. The CNN outputs each of two classes: boundary of sinus (*p*_1_) or lesion (*p*_2_). In the interactive step, we use the probability value of two classes to give speed compensation, which is calculated using the following equation based on the MS model:

(7) $$\begin{array}{c} \frac{\partial \phi }{\partial t}\left(x\right)=\delta \phi \left(x\right)\int_{\Omega_{y}}B\left(x,y\right)\delta \phi \left(y\right)\cdot \left(\lambda 1\frac{{\left(I\left(y\right)-v_{x}\right)}^{2}}{A_{v}}-\lambda 2\frac{{\left(I\left(y\right)-u_{x}\right)}^{2}}{A_{u}}+\exp\left({\left|\lambda_{1}+\lambda_{2}\right|}^{1/2}\right)\left(\frac{1+p_{2}}{1+p_{1}}-\frac{1}{2}\right)\right)\text{d}y+\mu \delta \phi \left(x\right)\text{div}\left(\frac{▽\phi \left(x\right)}{\left|▽\phi \left(x\right)\right|}\right), \end{array}$$

where *λ*_1_ impacts the weight of moving the curve outward along its normal and, alternatively, *λ*_2_ tries to move the curve inward. Both relative items interact on each other and decide ultimate magnitude of speed. exp(|*λ*_1_+*λ*_2_|^(1/2)^)(((1+*p*_2_)/(1+*p*_1_)) − (1/2)) is the term of speed compensation. Our research relies on an important assumption that any initial contour is given inside sinus cavity, since there are plenty of other sinus components outside bringing with impossibility to automatic segmentation. For a stable point, if *p*_2_ ≫ *p*_1_, it can be paid for a great of velocity and tend to move far away. If it locates in the region of the maxillary sinus boundary, *p*_2_ ≪ *p*_1_, the compensation is nearly zero, maintaining current status till convergence. We drop the confused condition, |*p*_1_ − *p*_2_| ≤ 0.2, without considerations in order to make sure of stability, which is found in very few cases in validation experiment.

#### 2.2.2. CNN Architecture

For the research of medical image segmentation, the most unsatisfied results are caused by heterogeneous texture feature that leaves the energy functional in local noisy minima. Our method generalizes this problem into a machine learning process with a common architecture for the CNN.

**Figure 3:**
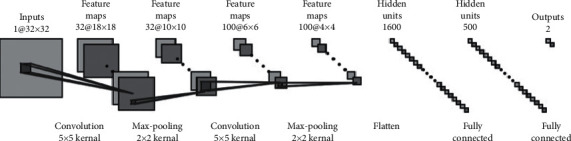
CNN architecture. The input is an 32 × 32 image of a point's local region. Two convolutional components include a 5 × 5 filter and 2 × 2 max pooling. Three fully connected layers contain 1600, 500, and 2 nodes, respectively.

**Figure 4:**
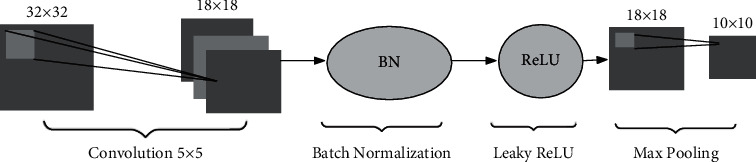
An instance of 1st convolutional block with batch normalization, Leaky ReLU, and max pooling.

The proposed architecture consists of two convolutional layers followed by three fully connected layers with outputs of two classes (Figure 3). The input of the image is sampled by a point's local region, and its size is fixed on 32 × 32. There are two convolutional layers at the beginning of our proposed CNN architecture, which includes a 5 × 5 filter and a 2 × 2 max pooling filter, respectively. The difference between two layers is the depth and stride. All hyperparameters we prefer have been tested to perform better than others. A nonliner activation function is applied to the outputs of the convolutional layer. In this paper, we choose Leaky ReLU [36] for network to give a sparse quality decreasing computing complexity and keeping backward propagation running smooth. To avoid overfitting and increase accuracy of outputs, we design a batch normalization (BN) filter [37] before ReLU configuration, which also resolves the problem of different distributions in training set or prediction. Each convolutional block of our CNN is illustrated in Figure 4. We give this light model as considerations of computation speed and the weight of our proposed program. A more complicated CNN should be offered, but it will affect the efficiency of the level set model that accounts for more computation costs.

#### 2.2.3. Training the CNN

In the project, the cost function of CNN is selected as cross-entropy loss, which evaluates the performance of the network after each batch:

(8) $$\begin{array}{c} L_{\text{loss}}\left(t,f\left(\omega ,x\right)\right)=-\frac{1}{N}\sum_{n=1}^{N}\overset{M}{\underset{m=1}{\sum }}T_{m,n}\log\left(p_{m,n}\right)+\frac{\eta }{2}{\left\|\omega \right\|}^{2}, \end{array}$$

where *t* represents the true label for every training example and *f*(*ω*, *x*) is the prediction function. When the *n*_th_ example is classified into *m*_th_, *T*_*m*,*n*_ equals 1. Otherwise, *T*_*m*,*n*_ equals 0. *p*_*m*,*n*_ is the probability of the *n*_th_ example being classified into the *m*_th_ class. In order to prevent overfitting, L2-regularization is introduced to penalize the size of the weights in *f*(*ω*, *x*), where *η*=1.0 is the coefficient of regularization. In the model parameters' learning, we adopt the strategy of moving average, including BN filter and stochastic gradient descent (SGD), and decay is set as 0.9997. With the same decay factor, for searching global minima effectively, every 10000 training steps, we adjust the learning rate exponentially based on the number of steps. And, in fully connected layers, we set dropout and keep probability 0.6 to make sure a sparse network for an acceptable result. To the initialization of model parameters, truncated Gaussian weight distribution is used for generation and standard deviation is 0.1. The CNN is trained with minibatch stochastic gradient descent with a batch size of 32 images. Toolkit of experiments depends on SLIM, a high level encapsulation of Tensorflow.

### 2.3. Implementation Details

#### 2.3.1. Without Reinitialization of Level Set and Narrow Band

Conventional level set has a boring drawback that the necessary signed distance function (SDF) cannot maintain special characteristic after some iterations. Common solution relies on reinitialization recurrently which costs plenty of computation time and produces numerical errors frequently. Li et al. come up with a regularization idea to resolve this problem [38] effectively. By appending a SDF regularization term on objective energy functional, the evolutionary process of zero level set contour is able to keep quality of SDF without reinitialization so that the complexity of algorithm has been reduced significantly and the final result is prominent among the peer. To save more computation time, the proposed method calculates the energy functional only for grid points located within a narrow band of the distance map, since localizing region-based level set [16] focuses on the local interior and exterior of points on the zero level set contour. Besides, through experiments, we found that the window size of these points outperforms nearly less than 10, a really ‘narrow' band ROI.

#### 2.3.2. Training Set and Image Preprocessing

**Figure 5:**
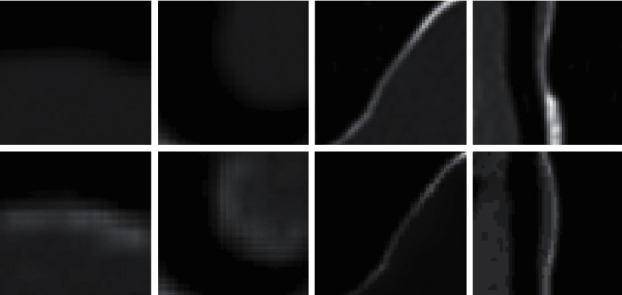
Examples of two classes and preprocessed inputs. The two columns on the left show images that were obtained on lesion edge and related preprocessed ones. The two columns on the right are from the bone of sinus cavity. All sampled points locate in the center of images with 32 pixels. By CLAHE analyzing, inputs of different classes stress their obvious features, assisting the CNN network a better performance of learning and predication.

The training set of two classes is divided into lesion or not. As we use CNN to analyze the static point's local region feature, it certainly stays at the edge of sinus cavity or lesion. Consequently, in detail, when constructing the training set, we sampled points on both conditions in average. The size of example is 10 × 10 and amplified to 32 by bilinear interpolation. During experiments, we tried different sizes and this group performed outstandingly. The number of training patches with two classes is collected as 50 thousands, respectively. For inputs of neural network, normalization of gray values is necessary for speed and accuracy. Common strategies are often influenced by noises greatly and insensitive to significant feature. We apply contrast-limited adaptive histogram equalization (CLAHE) that enhances two classes' different texture feature expressions and reduces interference of trivial noises.  Figure 5 shows a subset of the training set and corresponding preprocessed feature maps.

#### 2.3.3. Data Augmentation

For enlarging the limited training set, the augment dataset was created by applying a combination of elastic and affine distortions. We created elastic distortions by generating stochastic displacement field with values within the range of [−1,1], convolving these fields with a range of Gaussian filters, and multiplying the resulting matrices by a range of constant factors, controlling the intensity of the deformation. We also tried to adopt rotation to augment training batches. The result showed this approach is limited in improvement of training quality. Therefore, we did not introduce this design at last.

## 3. Results and Discussion

### 3.1. Dataset

In our study, approved by an institutional review board for restricted domain in our project, we used 50 CT volume scans (12.13 GB) by SOMATOM definition AS + SIEMENS containing maxillary sinus to evaluate the proposed multitask network BE-FNet. All of them have the same 512 × 512 in-plane resolution but with a different number of axial slices. The spacing between pixels along ZYX axes of the acquired dataset falls within from 0.5 × 0.35 × 0.35 mm to 0.625 × 0.39 × 0.39 mm. We analyzed 660 CT images of maxillary sinus with lesions as training and validation sets. Another 200 images were selected as the testing set with all possible cases. The following image acquisition parameters were used: manufactured by SIEMENS, SOMATOM definition AS+; 120 KVP; 500 mm data collection diameter; pixel spacing of 0.390625 mm; thickness of 0.6 mm; 15.2784 CTDvol. All CT images have isotropic pixels.

In the Section 1, we demonstrated that the size, location, and heterogeneity of lesion have high diversity of characteristics. A wide extent was found in the full set of images. Therefore, using a fixed group of parameters in energy functional for all cases of maxillary sinus is not feasible in practice. In other words, a novel solution should be supplied to stop points from falling into local minima. A wide range of lesion's intensity distribution illustrates the importance of and need for speed compensation based on convolutional neural network that evaluates feature of regions and achieves global optimum.

For evaluation, two radiologist supplied annotations of manual segmentation as ground truth with more than five years of experience. The final result of our proposed method was quantitatively compared with the average of the two radiologists' marking. For the research on sensitivity of segmentation to varied initializations, in every image, we gave five radii 3, 5, 7, 9, and 11 pixels. All initial contours are located inside cavity of sinus. Some were close to the center and others near the sinus boundary. This broad range of initializations allowed us to evaluate robustness of our algorithm in any case.

### 3.2. Segmentation Performance

**Figure 6:**
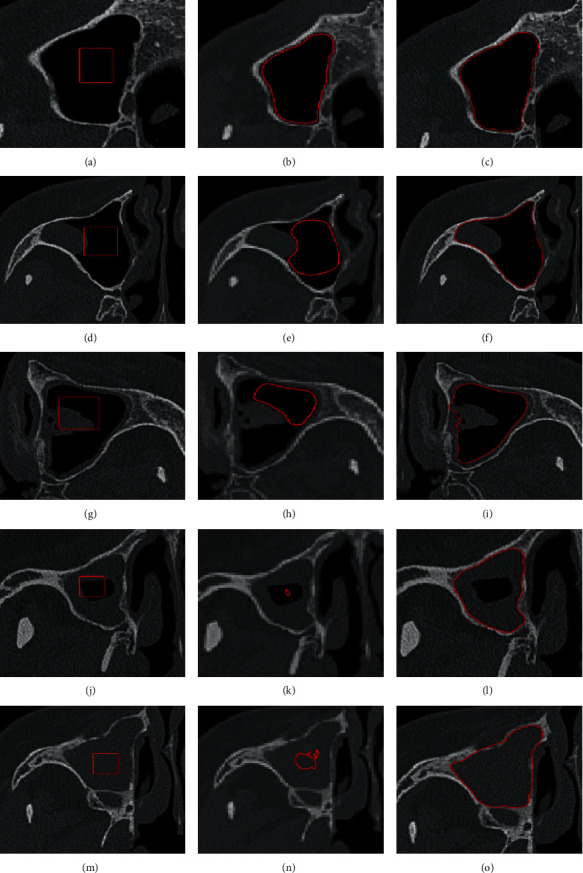
Comparison of FLS with our proposed method. (a, d, g, j, m) Some cases of maxillary sinus with different cases of lesion and the same initial contours. (b, e, h, k, n) Unacceptable results with FLS. (c, f, i, l, o) Predominant outcomes by our proposed method.

Energy functional of parameters *μ*_1_=0.05, *λ*_1_=1.0, and *λ*_2_=1.0 was used, as we tested them with the best performance for all 660 CT images without speed compensation. Figures 6(c), 6(f), 6(i), 6(l), and 6(o) show some examples of segmentation for different cases. Segmentation performance was assessed using the Dice similarity coefficient (Table 1). The Dice coefficient was calculated relative to each radiologist's manual marking, and then, an average Dice score was estimated. Our proposed segmentation method has high agreement with the manual markings for different local energy models. Results were better than the overlap that was measured between the complete manual annotations of both radiologists, thus demonstrating the strength of our proposed method.

### 3.3. Comparison with Fixed Contour Parameter Method

We compared our method with a state-of-the-art work energy model of localizing region-based level set based on fixed parameters [16] (FLS). In this experiment, we chose *μ*_1_=0.05, *λ*_1_=1.0, and *λ*_2_=1.0 as parameters of energy functional, since they had the best performance in all training set without speed compensation.  Figure 6 shows some cases of different maxillary sinus, initial contour, and final segmentation of the object, using both our proposed method and FLS. The left column supplies a series of CT images with lesions that have uncertain sizes, intensities, and locations obviously. Therefore, FLS has higher probabilities to induce the initial contour trapped into edge of lesions. The right column proves our method's advantage.

**Figure 7:**
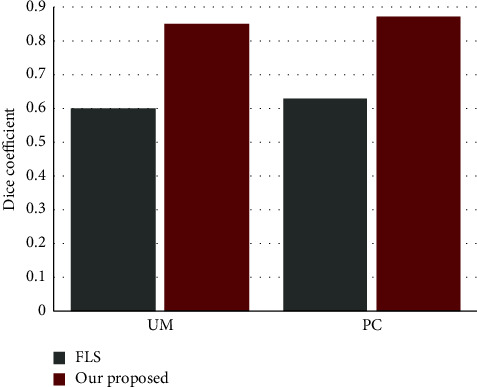
Average dice coefficient for two methods and 90% confidence interval for maxillary sinus segmentation over five contour initializations.

For quantitative evaluation two method, we acquired another 200 CT images as testing set. And, the Dice coefficients were averaged on five initial contours. The statistics shows that FLS has average Dice coefficients 0.60 ± 0.12 and 0.63 ± 0.10 for the UM and MS model, respectively. These Dice coefficients were significantly lower than the proposed method.  Figure 7 clearly shows that our method outperforms the state-of-the-art FLS.

**Figure 8:**
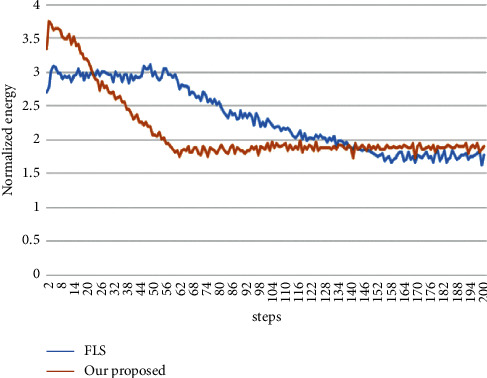
Convergence of energy functional on FLS and our proposed method.

Figure 8 demonstrates the convergence of FLS and our proposed algorithm on iterations of energy functional. At the beginning of running, both of them had a tendency to increase a little and FLS's extent more great. As expected, energy decreased with increasing iterations, converging on a single value; this implies minimization of the energy functional. For both metrics, substantial convergence was obtained after more than 60 iterations. Obviously, our proposed method had a faster speed of convergence. If adjusted to a proper larger length of the step, both of energy functional needed fewer times of iterations.

### 3.4. Comparison to Other State-of-the-Art Methods

We compared ours to another state-of-the-art approach proposed by Kamnitsas et al. [32]. The author presented a novel model integrating fully conventional network (FCN) and conditional random field (CRF) for segmentation of the medical image. This paper devised an cost function which joined CRF item to describe more accurate classes of pixels in neighborhood, removing false positive effectively. In addition, they employed a dual pathway architecture that processes the input images at multiple scales simultaneously for accurate localization of the object. Their innovative works improved segmentation skill based on full deep learning and won growing attentions.

We tested Kamnitsas' method (CRF-FCN) of 2D mode on the same training or testing datasets. As CRF-FCN is fully automatic, it did not need any initial contours. On configuration of CRF-FCN hyperparameters, we followed the authors' idea including 50 trees and maximum depth of 30 in random forest baseline. CRF-FCN supplied 90% confidence interval of 0.75 ± 0.12 with a large distribution of Dice coefficients. These results enhance the strength of our proposed method, which is significantly better than both FLS and CRF-FCN.

**Figure 9:**
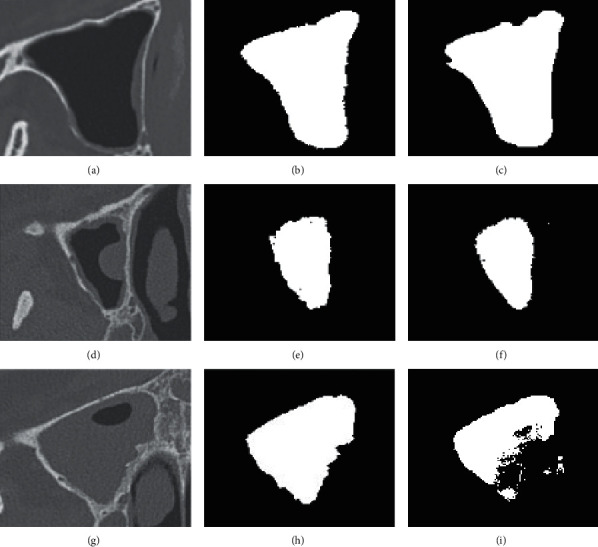
Examples of CRF-FCN methods. For explicit expression, masks had been introduced. The left column represents original CT images with lesions of different cases. Images of the middle column are ground truth of manual segmentation. The right column is the result processed by CRF-FCN.

Figure 9 illustrates results of the CRF-FCN method, performances of which had completely different accuracies in a large extent. To sinus with little lesion, it seemed qualified for segmentation. In the third row, a case filled with plenty of lesions failed to predict segmentation of dense pixels. In the research, we found that the performance of CRF-FCN or similar deep learning skills depends on whether the class distribution of the training set is balanced. If cases of specific pattern are seldom or redundant, they definitely lead the CNN network to stress or ignore corresponding features. Consequently, setting up a class-balanced training set is crucial but a big challenge for practice because of the large extent of space distribution for maxillary sinuses. Moreover, at the end of CRF-FCN, there is a layer of the CRF model for refinement. It takes more considerations on a pair of pixels with similar position and intensity into the same class. Most of lesions hold approximated features with neighbor regions outside sinus or boundary of cavity, which also has a negative influence on segmentation. For the testing set, completely different distributions of class in maxillary sinus such as size, abnormal structure, and imaging level definitely causes a relative lower accuracy. So, if there is another sufficient testing database, it should have a chance of improvement in evaluation. In a word, independent identical distribution (IID) of training or testing plays an important role especially in segmentation based on the neural network model.

### 3.5. Computational Time

We examined the computational time required by the proposed method to analyze segmentation. All data were trained and predicted using Tensorflow 1.2, and the process of level set happened in MATLAB R2017b 64 bit on Mac OS. Compared with FLS, our method needs an additional procedure of estimation for points till convergence of energy. In addition, the duration of one prediction for stable points on the zero level set by CNN was around 0.2s. In statistics, our proposed method costs 30% longer than FLS on average.

### 3.6. Sensitivity to Initialization

Different initializations of contours have great impacts on FLS [39, 40]. In the experiment, we evaluated FLS and ours on five different initial contours on which total Dice coefficients were averaged (Table 2). Our proposed method showed better agreement with the manual marking and smaller changes in the segmentation performance when it was applied using different energy models, significantly better than FLS. The result revealed that despite with noisy lesions, our model can deal with substantial deviations of the location of the initial contour, holding great robustness.

## 4. Conclusions

Segmentation of maxillary sinus with lesion faces a problem that size, location, and heterogeneity are seriously irregular so that the state-of-the-art method cannot survive. Most of them are based on gray gradient which result in the active contour falling in local minima of lesion edge. How to escape and arrive the object is the key of our research.

In this paper, we present a novel method of an adaptive localizing region based of the level set using convolutional neural network. The algorithm automatically analyzes the region feature of a stable point on the active contour in every iteration. If it locates in the lesion, its speed could be compensated based on the probability of outputs by CNN. If not, it stays still till the end of iteration. The proposed mechanism makes sure the trapped point could evolve outward. Therefore, our method is adaptive to cases of sinuses with possible lesions. Our proposed method shows high agreement with expert manual marking for a diverse dataset of CT images. The variety of spatial texture characteristics in our datasets emphasized the strength of our adaptive method, which performed well with inhomogeneous lesions and with noisy backgrounds.

We compared our results to uniform modeling (UM) and mean separation (MS) models of local version [16]. With the testing set of 200 images, Figure 6 demonstrates that fixed parameters of the localizing region-based level set cannot resolve problems, and most contours stop in local minima, especially illustrated in the cavities filled with more lesions. Moreover, our method has obvious predominance over FLS and is confirmed by statistics on quantitative evaluation (Table 2).

Kamnitsas' method (CRF-FCN) [32] is very popular recently and derived from fully convolutional network and conditional random field ideas. It has been proved an efficient and effective algorithm compared with the other state-of-the-art method. With contrast experiment, our proposed method also outperformed significantly and drawbacks of CRF-FCN had been stated. Great variations of appearance in maxillary sinus denote CRF-FCN's frustration furthermore.

Besides, we discussed the convergence of FLS and our method. Figure 8 shows that ours has a faster speed of convergence although it costs 30% more longer computation time on an average. Meanwhile, experiments also gave the confidence of insensitivity to initialization of contours. It implies a great potential of our method in full automatic segmentation. Consequently, our combination of deep learning and level set captures the benefits of both approaches and overcomes their limitations, to achieve significantly better results than either method alone.

The presented work has some limitations. First, whether *μ*_1_, *λ*_1_, and *λ*_2_ of energy functional are sensitive to our method is not discussed in the work. In addition, 3D segmentation based should be a future direction, as well as incorporation of full automatic segmentation, without any user input. In summary, the method presented shows more advanced skills compared with the state-of-the-art level set methods. It performed better in heterogeneous texture between foreground and background, providing a new area of research.

## Figures and Tables

**Table 1:** Average dice coefficient and 90% confidence interval (CI) for the maxillary sinus segmentation using our proposed method based on CNN estimation.

	Our proposed method vs. ground truth
UM model	0.85 ± 0.05
MS model	0.87 ± 0.04

**Table 2:** Average dice coefficient and 90% confidence interval (CI) for the maxillary sinus segmentation, comparison of our proposed method and FLS.

Our proposed method vs. FLS		
	Our proposed	FLS
UM model	0.85 ± 0.05	0.60 ± 0.12
MS model	0.87 ± 0.04	0.63 ± 0.10

## Data Availability

The experimental data used to support the findings of this study are available from the corresponding author upon request.
